# *Arabidopsis* F-BOX STRESS INDUCED (FBS) proteins have distinct subcellular interaction patterns with 14-3-3 proteins

**DOI:** 10.17912/micropub.biology.002221

**Published:** 2026-08-31

**Authors:** Mael Bradley, Meghan Bacher, Kaitlin Riggan, Nicolo Torresan, Megan Tegman, Sabine Angier, Amy Replogle, Bryan Thines

**Affiliations:** 1 University of Puget Sound, Tacoma, WA; 2 University of California, Berkeley, CA; 3 Washington State University, Pullman, WA; 4 University of Rhode Island, Kingston, RI

## Abstract

F-BOX STRESS INDUCED (FBS) proteins are substrate adaptors in SCF-type E3 ubiquitin ligases that regulate plant development and stress responses. *Arabidopsis thaliana* FBS1 (AT1G61340) interacts with multiple 14-3-3 proteins, but it is unknown whether other FBS family members share these interactions. Bimolecular fluorescence complementation (BiFC) assays in *Nicotiana benthamiana* showed that all four FBS proteins interact with multiple 14-3-3 proteins in vivo. However, FBS1 interactions were restricted to the nucleus, whereas FBS2 – FBS4 (AT4G21510, AT4G05010, AT4G35930) interactions were detected in both the nucleus and cytosol. These findings indicate conserved 14-3-3 interactions within the FBS family, but suggest functional divergence in subcellular interaction profiles.

**Figure 1. Arabidopsis FBS interactions with 14-3-3 proteins in bimolecular fluorescence complementation (BiFC) assays:** **(A – D)** nYFP-FBS1 through nYFP-FBS4 were co-expressed with cYFP-14-3-3 ω and H2B-RFP in four-week-old *N. benthamiana* leaves. Cell walls were stained with 50 μM propidium iodide (PI), and images were taken three days after agroinfiltration. BiFC (green; first column) indicates protein interaction, while H2B-RFP and PI staining (red; second column) show nuclei and cell walls, respectively. Merged images (third column) show co-localization of BiFC signal with nuclei. **(E)** nYFP-PP2-A12 was co-expressed with cYFP-14-3-3 ω and H2B-RFP as a negative control. **(F – J)** The same nYFP fusion constructs and H2B-RFP were co-expressed with cYFP-14-3-3 ψ under identical conditions. **(K – N)** nYFP-FBS constructs were co-expressed with cYFP-SPA1; the BiFC channel is shown (all channels are shown in Extended Data). Scale bar = 100 μm. **(O)** Average number of nuclei exhibiting BiFC signal per field of view (FOV) for nYFP-FBS family members co-expressed with cYFP-14-3-3 ω, and **(P)** co-expressed with cYFP-14-3-3 ψ. Error bars represent SE (n = 3 independent experimental replicates). Statistical significance was assessed using one-way ANOVA followed by Tukey’s HSD post-hoc tests. Means with different letters are significantly different from each other at *P* < 0.05.

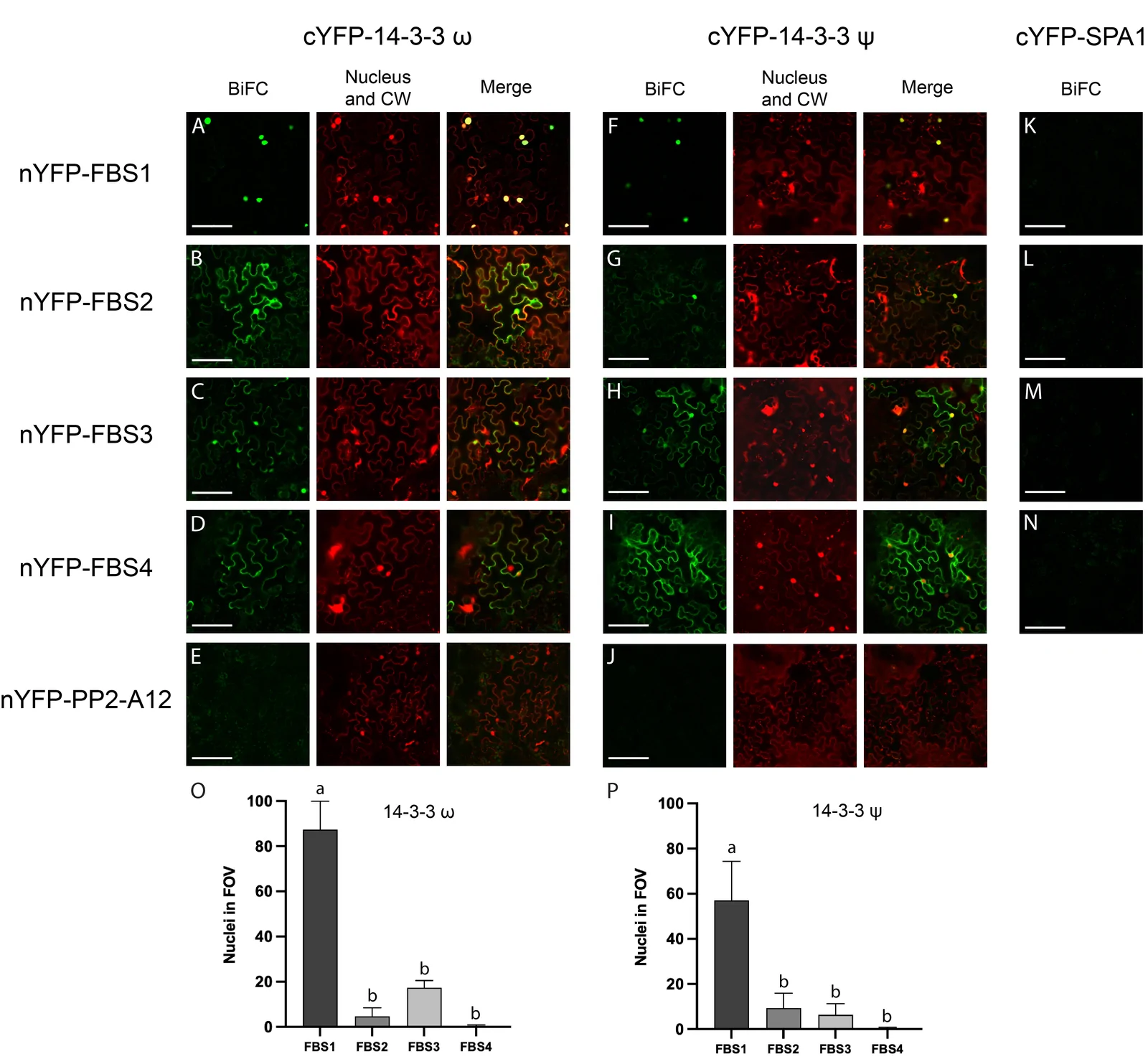



## Description

Numerous cellular pathways in eukaryotes are regulated through selective protein degradation by the ubiquitin 26S proteasome system (UPS). SKP1-CUL1-F-box (SCF) complexes are a prevalent type of E3 ubiquitin ligase in the UPS that use F-box (FBX) proteins as substrate adaptors to specifically recruit ubiquitination targets (Lee et al., 2018; Sheard et al., 2010; Varshney et al., 2026). Four *Arabidopsis thaliana* F-BOX STRESS INDUCED proteins (FBS1 – FBS4) compose a subfamily of plant-specific FBX proteins (Maldonado-Calderon et al., 2012). FBS1 and FBS4 target anaphase-promoting complex/cyclosome (APC/C)-associated regulatory proteins to control cell division events and related processes, including maintenance of the root quiescent center (QC) and cell fate determination during stomatal development (Geem et al., 2022; Li et al., 2022; Zhang et al., 2025). FBS proteins also likely target two other nuclear-localized WD40 repeat-like family proteins of unknown function (Sepulveda-Garcia et al., 2021).

In addition to its targeting interactions, FBS1 interacts with at least six of thirteen proteins belonging to the Arabidopsis 14-3-3 family in yeast two-hybrid and in vitro pull-down assays (Sepulveda-Garcia & Rocha-Sosa, 2012). 14-3-3 proteins are eukaryotic regulators that specifically interact with their client proteins to alter their activity (Wilson et al., 2016). While some 14-3-3 proteins are SCF targets in plants (Hong et al., 2017), FBS1 does not seem to target 14-3-3 proteins for degradation, as increased FBS1 abundance does not lead to a decrease in the level of FBS interactor 14-3-3 λ (AT5G10450) (Sepulveda-Garcia et al., 2021; Sepulveda-Garcia & Rocha-Sosa, 2012). Furthermore, 14-3-3 proteins interact with FBS1 via the F-box domain (Sepulveda-Garcia & Rocha-Sosa, 2012), which is required for FBX protein interaction with Skp and the core SCF complex. The regulatory purpose and biological consequence of FBS1 interaction with 14-3-3 proteins are currently unknown.

Members of FBX protein subfamilies often have some functional redundancy while still maintaining distinct biological roles (Lee et al., 2018). FBS1 – FBS4 likely have distinct biological functions relative to each other based on their expression profiles and mutant phenotypes (Geem et al., 2022; Li et al., 2022; Maldonado-Calderon et al., 2012). However, it is unknown whether FBS2 – FBS4 also interact with 14-3-3 proteins and whether they might experience similar regulatory mechanisms to FBS1. Moreover, while FBS proteins interact with at least some of their targets in the nucleus (Li et al., 2022; Sepulveda-Garcia et al., 2021), the subcellular locations of FBS interactions with 14-3-3 proteins are unknown. Having this interaction information could offer clues as to how 14-3-3 proteins work with FBS proteins within the regulatory network associated with the APC/C to influence plant growth and development. We therefore tested all four Arabidopsis FBS proteins for interaction with multiple Arabidopsis 14-3-3 proteins in living plants using bimolecular fluorescence complementation (BiFC) assays. 

Among the four members of the FBS protein family, distinct interaction profiles with 14-3-3 proteins were observed. FBS1 interactions with 14-3-3 ω (AT1G78300) and 14-3-3 ψ (AT5G38480) were restricted to the nucleus under the conditions tested, as shown by colocalization of the BiFC signal with nuclear-localized fusion protein H2B-RFP ( Figure 1 A and F). In contrast, FBS2 – FBS4 had fewer interactions with these 14-3-3 proteins in the nucleus, but they interacted in the cytosol ( Figure 1 B – D and G – I). The frequencies of nuclear interactions between FBS2 – FBS4 and 14-3-3 proteins ω or ψ were less than 25% of those found for FBS1 interactions in a field of view ( Figure 1 O and P). Because the F-box domain is required for FBS1 interaction with 14-3-3 proteins (Sepulveda-Garcia & Rocha-Sosa, 2012), we tested the phylogenetically related FBX protein PP2-A12 (AT1G12710), one of the two closest non-FBS FBX family members based on F-box domain sequence analysis (Gagne et al., 2002). In contrast to the FBS proteins, no BiFC signal was detected when PP2-A12 was co-expressed with either of the 14-3-3 proteins under the conditions tested ( Figure 1 E and J). As an additional negative control for nonspecific interactions, FBS proteins were tested with the unrelated nuclear-localized protein SPA1 (AT2G46340) (Zhu et al., 2008), and no detectable BiFC signal was observed for any of the four FBS proteins ( Figure 1 K – N, see also Extended Data). Collectively, these findings show distinct subcellular patterns of FBS/14-3-3 interactions in vivo, while the negative control results are consistent with specificity of the observed BiFC interactions.

FBS proteins have numerous protein interactors, some of which are ubiquitylation targets, while others may regulate FBX protein function and the ubiquitylation process. This work found that all four FBS proteins interacted with 14-3-3 proteins in living plant cells, indicating that 14-3-3 interaction is conserved within the FBS family. However, FBS1 showed the highest frequency of nuclear interactions with the 14-3-3 proteins under the conditions tested here, suggesting potential functional divergence or distinct contributions among family members. Although the negative controls are consistent with specificity of the observed FBS/14-3-3 interactions, PP2-A12 and SPA1 protein accumulation was not independently verified and alternative orientations of the YFP fragments were not tested. Thus, differences in protein abundance or steric constraints could contribute to the absence of BiFC signal with PP2-A12 or SPA1, and these results should not be interpreted as definitive evidence for lack of interaction. Moreover, BiFC can stabilize transient protein interactions (Kudla & Bock, 2016), and therefore these findings may not fully reflect endogenous interaction dynamics. Nevertheless, the absence of detectable BiFC signal between FBS proteins and SPA1 or between 14-3-3 proteins and PP2-A12, together with the distinct subcellular interaction patterns observed among FBS family members and previous yeast two-hybrid and in vitro pull-down evidence for FBS1/14-3-3 interactions (Sepúlveda-García & Rocha-Sosa, 2012), provides support for the specificity of the observed FBS/14-3-3 interactions.

The N-terminal region and F-box domain of FBS1 have previously been implicated in 14-3-3 binding (Sepulveda-Garcia & Rocha-Sosa, 2012), but whether these regions similarly mediate 14-3-3 interactions with FBS2 – FBS4 remains unknown. Canonical Mode I or Mode II 14-3-3 recognition motifs often mediate 14-3-3 protein interaction with the client protein (Camoni et al., 2018). None of the four FBS proteins contains either of these motifs. The absence of canonical recognition motifs suggests that FBS interactions with 14-3-3 proteins may involve noncanonical phosphorylation-dependent binding sites or an alternative mode of interaction. Sequence analysis using 14-3-3-Pred, software that predicts 14-3-3-binding phosphopeptides (Madeira et al., 2015), identified several Ser/Thr residues in FBS1, FBS2, and FBS4 with scores above the consensus prediction threshold, despite their occurrence outside canonical recognition motifs. These residues therefore represent candidate phosphorylation sites for future studies testing the effects of amino acid substitutions on 14-3-3 binding.

The purpose of FBS interaction with 14-3-3 proteins is currently unknown. One possibility is that these interactions regulate FBS distribution within the cell, as some 14-3-3 protein interactions control the subcellular localization of client proteins (Gampala et al., 2007; Huang et al., 2018). A more in-depth investigation of FBS localization, whether bound to 14-3-3 proteins or not, may clarify whether cellular distribution plays a role in restricting FBS activities. The roles of 14-3-3 proteins in regulating FBS localization could then be tested through genetic reduction of 14-3-3 activity in Arabidopsis mutants or through chemical inhibition using AICAR (Toroser et al., 1998). Alternatively, interactions between FBS and 14-3-3 proteins may have important implications for SCF complex assembly and substrate specificity. SCF complex dimerization can enhance substrate recognition capabilities and/or diversify target range (Tang et al., 2007; Welcker et al., 2013), and facilitating FBX dimerization is a role of some 14-3-3 proteins (Barbash et al., 2011). Considering FBX protein homo- and hetero-dimerization (Kominami et al., 1998), having four Arabidopsis FBS family members raises the possibility that combinatorial dimerization could expand target range as the number of verified SCF ^FBS^ targets continues to grow. The differential localization observed here raises the possibility that 14-3-3-mediated, compartment-specific FBS dimerization could contribute to distinct functions among FBS family members. Future work could therefore assess the effects of 14-3-3 proteins on SCF ^FBS^ target selection and ubiquitylation activity in both in vitro and in vivo systems.

Determining whether 14-3-3 proteins regulate FBS localization or enable SCF ^FBS^ substrate recognition may lead to a deeper mechanistic understanding of how plant stress responses are controlled. Several 14-3-3 proteins that interact with FBS proteins regulate abiotic stress signaling (Catala et al., 2014; Tan et al., 2016; van Kleeff et al., 2014). Likewise, both FBS1 and the rice homolog FBX257 are functionally connected to plant abiotic stress responses, either through transcriptional effects or mutant phenotypes (Geem et al., 2022; Gonzalez et al., 2017; Maldonado-Calderon et al., 2012; Sharma et al., 2023). Future work investigating how FBS and 14-3-3 proteins function together should therefore include conditions that specifically address environmental stress. Collectively, this work expands understanding of FBS/14-3-3 family-wide interactions and suggests future studies examining their mechanistic roles in environmental stress signaling.

## Methods

**Plasmid construction**

Standard molecular biology cloning protocols were used to generate Gateway-compatible constructs (Thermo Fisher Scientific). *FBS* and *14-3-3* coding sequences were amplified from cDNA by PCR with gene-specific primers (Table 1) using Phusion High-Fidelity DNA Polymerase (Thermo Fisher Scientific). Amplicons were cloned into the pENTR entry vector with the pENTR/D-TOPO directional cloning kit (Thermo Fisher Scientific). Genes were then transferred into either pCL112 (creates N-terminal fusion to nYFP) or pCL113 (creates N-terminal fusion to cYFP) with LR Clonase II enzyme mix (Thermo Fisher Scientific). Sequences were verified by Sanger sequencing (Eurofins Genomics).

**Agroinfiltration of *Nicotiana benthamiana* leaves**

*Agrobacterium tumefaciens* strain GV3101 pMP90 was transformed by electroporation and selected by appropriate antibiotics. Seed cultures were grown in liquid LB for 2 days with shaking at 28 ^o^ C. Full cultures were inoculated with seed culture at a 1:100 dilution and grown for 24 hours under the same conditions. Cells were pelleted and resuspended in infiltration medium (10 mM MES, 10 mM MgCl _2_ , 100 μM acetosyringone) and incubated for 5 hours with rocking at room temperature. Cells were pelleted a second time and resuspended in infiltration medium. Appropriate nYFP/cYFP pairs, H2B-RFP, and p19 suppressor strains were mixed at a final OD _600_ of 1.0 for each strain. The abaxial side of *Nicotiana benthamiana* leaves from 4-week-old plants grown under greenhouse conditions was infiltrated by syringe with the *A. tumefaciens* mixes using standard protocols (Leuzinger et al., 2013).

**Imaging**

Three days after infiltration, *N. benthamiana* leaves to be imaged were infiltrated with 50 μM propidium iodide in 0.1% Tween-20 and allowed to sit for 10 minutes. Approximately 1 cm ^2^ from infiltrated leaves was then cut and mounted on a glass slide with 0.1% Tween-20. The underside of whole leaf mounts was visualized by laser-scanning confocal microscopy using a Nikon D-Eclipse C1 Confocal laser scanning microscope (Nikon Instruments) with either: 1) excitation at 488 nm with an emission band-pass filter of 515/30, or 2) excitation at 561 nm with an emission band pass filter of 650 LP. Images were processed with Fiji software (http://imagej.net/software/fiji/). Representative images from at least three independent experimental replicates are shown.

**Data analysis**

Nuclei containing a BiFC fluorescence signal were counted under a 20x objective lens in two separate areas of each leaf, which were then averaged, from three independent experimental trials. The field of view (FOV) diameter was 1100 µm. Statistical analyses were performed in RStudio version 4.0.4 using a one-way ANOVA and Tukey’s HSD post-hoc test.

## Reagents

**Table:** 

		**Primer sequences 5' to 3'**	
**Gene**	**AGI number**	**Forward**	**Reverse**
*FBS1*	At1g61340	CACCATGGCATTGGGGAAGAAAAGAATCG	TCAGTGGAATAGAGCCACTGAGAC
*FBS2*	At4g21510	CACCATGATCCATTATCTCCATTTCA	TCATGTAAACAAAGCCGCAG
*FBS3*	At4g05010	CACCATGGCGTATTTGAGTGATG	TCATTTAAACAATACCATAGAGATCTTCGACAAATC
*FBS4*	At4g35930	CACCATGGGGAAGGTATCTCCAAAG	TCAGGTGAGGTTGTTTTGAGC
*PP2-A12*	At1g12710	CACCATGGGTGTGGCTCACTCTGAT	TTAAAACCGCTTCAACTGGTC
*14-3-3 omega*	At1g78300	CACCATGGCGTCTGGGCGTGAAG	TCACTGCTGTTCCTCGGTCG
*14-3-3 psi*	At5g38480	CACCATGTCGACAAGGGAAGAGAATGT	TTACTCGGCACCATCGGG

## Data Availability

Description: Arabidopsis FBS proteins co-expressed with SPA1 in bimolecular fluorescence complementation (BiFC) assays. Resource Type: Image. DOI: https://doi.org/10.22002/mpexm-85m05
